# Fake news in COVID-19: A perspective

**DOI:** 10.34172/hpp.2020.44

**Published:** 2020-11-07

**Authors:** Diego Carrion-Alvarez, Perla X. Tijerina-Salina

**Affiliations:** Basic Science Department, School of Health Sciences, Universidad de Monterrey, Av. Morones Prieto 4500 Ote. Jesús M. Garza. San Pedro Garza García, C.P.66238 Nuevo León, México

## Dear Editor,

Being a health professional during this pandemic crisis has been more than overwhelming. As of August 15th, COVID-19 has infected more than 21 million people and caused the death of more than 755 000 patients worldwide.

As health professionals, we have always faced “Fake News” in our professional practice, from alternative treatments to conspiracy theories like “Big Pharma”, and the most popular: vaccines causing autism. With COVID-19, the amount of Fake News has increased substantially, and has generated situations that negatively impact public health in multiple regions of the world. We are not only struggling with the virus but with an important infodemic based on ignorance and misinformation.

## Anti-vaxxers and Fake News

The burden of misinformation has been exposed worldwide during this pandemic; manifestations have varied drastically within different regions, however, one type in particular is global: the anti-vaccine manifestations. This movement started around 1998 after Andrew Wakefield’s paper associating autism with vaccines, and despite the retraction of the article from The Lancet and the scientific demonstration of vaccine security, the anti-vaxxer movement continues to strengthen.^1^ The use of social media and diffusion in videos and supposed evidence in platforms such as YouTube has been key in the propagation of the infomedia.^2^ This behavior represents an important threat to public health, as stated by the World Health Organization (WHO) in 2019. During this pandemic, these destructive beliefs have continued if not increased: according to a survey conducted in Europe, approximately 10% of the interviewed population in Germany and France declared that they will not accept a COVID-19 vaccine; France had the highest percentage of “unsure” with 28%.^3^ Anti-vaccine rallies have been popular in countries like the United States and certain regions of Europe. Additionally, manifestations against lockdowns and obligatory mask use have emerged in several countries such as Canada, Germany, Poland, the United Kingdom, and the United States, among others. Furthermore, in some countries, the consequences of these fake news include shortages of medicines such as hydroxychloroquine or medical facial masks. In Europe, patients refused to take ibuprofen after some viral misinformation claimed it worsened the COVID-19 symptoms.^4^ It even contributed to increased racism against Chinese individuals in several regions of the world such as Japan or the United States.^5^

Conspiracy theories have also been added to the mix, ranging from the well-known “Big Pharma” and the non-existence of the virus to claims of microchips in vaccines, the stealing of personal information, and the implementation of 5G to decimate the population.^6^ This wide range of misleading statements has resulted in people ignoring social distancing and other preventive health measures, as commonly seen in social media posts.^7^

## Public figures and violence

Public figures such as celebrities, news presenters, and politicians play a crucial role during the pandemic; while they are only responsible for 20% of the misinformation, they represent 69% of social media engagement.^8^ Several presidents such as United State’s Donald Trump, Mexico’s Andres Manuel López Obrador and Brazil’s Jair Bolsonaro have spread false information such as the safe injection of disinfectants, undermining the importance of facemask use and overall contradicting medical advice, going as far as outright denying the pandemic. It is important to recognize that they are, inevitably, role models, and their actions can negatively impact the population. Unfortunately in some countries, the manifestations against health measures have not been limited to opinionated social media posts and peaceful protests. In Mexico, there have been reports of health personnel having hot coffee or bleach thrown at them on the streets, as well as attempts to burn their homes, and even beatings of nurses and doctors; in some towns, the habitants have even arranged to burn COVID-19-designated hospitals. These kinds of incidents have also frequently been reported in India.

As the pandemic grows worldwide, we are not only dealing with the virus and its capacity to overwhelm healthcare institutions, but also with a wave of misinformation that is costing human lives and negatively impacting those who work for the health and wellbeing of the population.^9^ This situation has also shed light on the need for educating both the population and the media, and has exposed prejudices against the medical community.

Several actions have already been taken to oppose the infodemia; the WHO has increased their efforts in monitoring social media platforms and has collaborated with Instagram, Facebook and Twitter to develop links to official pages any time someone searches for “COVID-19” or “coronavirus”.^10^ Moreover, governments around the world such as Iran have worked on strategies looking to empower the people so they can properly inform the rest of the population and contribute to recovering public trust.^11^ We are still far from a flawless model that raises the voice of scientists and scientific associations in social media, and as long as the efforts to stop the infodemic remain uncoordinated, we will not be able to overcome it. This crisis is also an opportunity to think about how we have been communicating and how we can improve.^12^ As health professionals we can contribute to tackle the infodemic with everyday actions such as sharing verified content in our personal and professional social media, listen to and answer our patients’ doubts, and educate our friends and family. If we all take small steps, we can help eradicate this infodemic and prevent the next one.

## Competing interests

The authors declare that they have no competing interests.

## Ethical approval

Not applicable.
